# Contemporary Research Questions in the Field of Contact Lenses and Where to Next?

**DOI:** 10.1007/s44402-026-00142-z

**Published:** 2026-07-22

**Authors:** Sukanya Jaiswal, Fiona Stapleton

**Affiliations:** School of Optometry and Vision Science, UNSW Sydney, Sydney, New South Wales Australia

**Keywords:** Biosensing, Contact lens, Drug-delivery, Myopia control, Theranostics, Visual augmentation.

## Abstract

This review paper provides an overview of contemporary research areas in the field of contact lenses, recent progress and outstanding questions. Beyond the introduction of silicone hydrogel materials and current widespread use of daily disposable soft lenses, material and design innovations over the past decade have expanded the scope of contact lens applications to include preventative strategies for myopia, drug delivery, biosensing, theranostic (diagnostic sensing and delivery of therapy in response), visual augmentation and communication. Material and nanotechnology innovations have supported drug loading and controlled drug release from contact lenses, using molecular imprinting, layer-by-layer assembly and the incorporation of colloidal and polymeric nanoparticles. Other than many optical interventions for myopia control, most applications are either in pre-clinical or clinical testing. Drug delivery and theranostic applications are likely to be advanced with standardisation of protocols and agreed outcome measures in clinical trials to support comparisons between studies and regulatory approval pathways. Development of visual augmentation, sensing and communication applications will depend on demonstration of clear benefits, affordability, comfort and wearer acceptance. While innovations beyond myopia control are largely not commercially available, the next decade is likely to translate these new technologies into broader use.

Key Points

- Contact lenses are being developed for use beyond refractive correction into a therapeutic and diagnostic device.
- Clinical translation and commercial adoption remain major barriers despite substantial technological progress.
- Myopia control remains the most clinically established advanced application of contact lenses, although questions regarding long-term safety, discontinuation and regional prescribing variability persist.

## Introduction

To provide some context to the current landscape of contact lens wear, silicone hydrogel contact lens materials were introduced 25 years ago [1], and these materials are currently prescribed three times more often than hydrogels. Daily wear soft contact lenses are used by 86% of wearers internationally, 45% of all new contact lens fittings are daily disposable and rigid corneal lenses, while orthokeratology lenses comprise around 10% of new lens fittings [2]. Challenges to increase the uptake of contact lenses for the correction of refraction include retention of existing wearers, management of contact lens discomfort and dryness, competition from surgical means of vision correction and a recognition that step changes in innovation are not expanding the pool of wearers.

Beyond the correction of simple refractive error, broader use of contact lenses may occur through expanding the possible applications for contact lens use. Advances in material technology, lens design and changes to wear modality have expanded applications in myopia control, drug delivery, biosensing, augmented reality and communication. This review will explore selected contemporary applications of contact lens wear and highlight important advances in the field.

## Approaches to Slow the Progression of Myopia

Myopia control applications initially involved soft multifocal contact lenses with varying power profiles and commonly with either peripheral addition or concentric rings of increased lens power. Peripheral myopic defocus is thought to generate an optical signal that limits eye growth. Optical and material advances expanded the range of soft contact lens materials, enabled daily disposable modalities and stimulated innovative coaxial and non-coaxial designs, including those to manipulate higher-order aberrations to improve visual quality while limiting axial elongation. In parallel, the use of orthokeratology, where rigid gas-permeable corneal lenses are worn overnight to temporarily reshape the cornea, has increased. While the central flattening corrects myopia during the day, the steepening of the mid-peripheral cornea induces peripheral myopic defocus, resulting in an optical signal to limit axial eye growth. This mechanism of peripheral myopic defocus may not fully explain the axial elongation reduction seen with multifocal soft contact lenses [3] or indeed with ring focus or centre near designs [4]. It is likely that other changes induced by myopia control strategies, including higher-order aberrations and retinal contrast, also guide eye growth.

In terms of performance and acceptability, studies in the last 10 years have suggested that for multifocal contact lenses, efficacy (measured as slowing axial elongation and myopia progression) is greater with higher addition multifocal lenses compared with lower additions and single-vision contact lenses, e.g., [5]. Reports from the International Myopia Institute regularly summarise the most recent evidence in controlling myopia onset and progression, and have reported greater reduction in axial elongation over 24 months with orthokeratology than with multifocal or extended depth of focus soft contact lenses [6]. A 2025 systematic review of discontinuation rates for myopia control therapies showed the lowest was amongst spectacle wearers, followed by orthokeratology, while soft contact lenses were associated with a higher likelihood of discontinuation than both of these modalities [7]. Compared with soft contact lenses, dropout was reduced by 71% in myopia control spectacle wearers and by 56% in those undergoing orthokeratology. Ocular adverse events and complications were identified as significant factors in discontinuation.

Safety data in paediatric populations using contact lenses remain limited. There are no adequately powered, population-based studies directly comparing complication rates between silicone hydrogel and hydrogel lenses in children. Aggregated data from eight prospective trials indicated that the incidence of infectious keratitis in paediatric soft contact lens wear was approximately 2.7 per 10,000 wearers per year (95% CI: 0.5–15), which is similar to adult rates for both daily reusable and daily disposable soft contact lenses [8]. Similarly, the incidence of corneal infiltrative events in paediatric wearers appears comparable to that observed in adults wearing contemporary soft contact lenses, at approximately 0.5% per year [8]. Rates observed in myopia control lens studies appear comparable to those associated with single-vision soft lenses, although interpretation is limited by small sample sizes and wide confidence intervals.

Similarly, the risk of infectious keratitis in paediatric orthokeratology has not been well characterised in prospective studies. Retrospective data from Japan and Moscow estimate an incidence of approximately 5.4 per 10,000 patient-years [9, 10]. While sight-threatening adverse events are uncommon, less severe complications such as corneal staining are reported more frequently in orthokeratology wear compared to soft lens modalities [11]. A Cochrane review on myopia control interventions stratifies adverse events by lens modality [12]. Papillary reactions, corneal infiltrates and erosions were the most common adverse events in myopia control studies using soft contact lenses, while corneal erosions were the most common adverse events reported in orthokeratology studies.

Perceptions of the efficacy of contact lenses for myopia control have also shifted substantially over the past decade. In a 2023 International Myopia Institute report based on a 9-year follow-up survey of 3000 eyecare practitioners worldwide, the perceived efficacy of myopia control soft contact lenses has increased [13]. The wide commercial availability of these designs, along with the efficacy, has driven an increase in the prescription of myopia control contact lenses in the past decade. However, regional prescribing patterns vary considerably. In 2024, orthokeratology was commonly prescribed for progressing myopes in Asia, whereas in Australasia and Europe, myopia control daily disposable soft contact lenses were prescribed with similar or greater frequency than orthokeratology. In contrast, single-vision soft contact lenses continue to dominate prescribing practices in the Americas [13]. However, this trend may shift following the 2025 US Food and Drug Administration (FDA) approval of the first myopia control spectacle lenses in the USA [14].

## Drug Delivery and Biosensing

### Theranostic Contact Lenses

Soft contact lenses have been shown to have theranostic potential, that is, the ability to simultaneously perform diagnostic biosensing and deliver controlled therapy. pH-responsive contact lenses, for example, exploit changes in tear fluid pH associated with inflammation or infection to trigger drug release [15]. However, more commonly, theranostic contact lenses have been designed to detect and manage ocular hypertension.

Contact lenses embedded with microstructure drug reservoirs are designed to be intraocular pressure (IOP)-responsive [16]. When IOP increases, corneal deformation compresses these microstructures, resulting in associated drug release. Ex vivo studies using porcine eyes demonstrated that manual fluctuation of IOP led to corresponding changes in drug release rates. In subsequent *in vivo* experiments, contact lenses loaded with timolol and brinzolamide were applied to rabbit eyes, producing a more sustained reduction in IOP compared to conventional topical eye drop administration.

IOP sensing within these systems can be achieved using electronic components, such as nanowires and embedded circuitry, to detect pressure changes and transmit signals that initiate drug release [17]. Alternatively, mechanosensitive IOP sensors have also proven effective, whereby IOP-induced displacement of liquid within microchannels triggers drug release [18]. IOP measurements with these sensors are comparable to those with gold standard tonometers. The size of the microchannels is associated with specific IOP thresholds, allowing customisation and precision care, as target IOPs in open-angle glaucoma management are based on individual risk factors. Notably, dual drug delivery via mechanosensitive contact lenses demonstrated a greater sustained reduction in IOP compared to single drug release, consistent with observations from topical therapy.

These innovative contact lens designs show significant promise for advancing glaucoma management and enabling personalised therapy. Human safety and efficacy trials represent the logical next step in this field.

### Drug-Delivering Contact Lenses

Over the past several decades, drug-eluting contact lenses have emerged as a promising alternative to conventional topical ocular drug delivery. Early efforts were motivated by the limitations of eye drops, including rapid drug loss from blinking and tear film turnover, high initial burst dosing and poor patient compliance with multi-dosing. These challenges prompted exploration of contact lenses as drug reservoirs capable of prolonging drug residence time on the ocular surface and enhancing bioavailability. Diseases that have been targeted include bacterial keratitis, dry eye, glaucoma, ocular allergy and ocular surface injuries such as chemical or physical trauma.

Initial approaches to drug loading used simple soaking of soft contact lenses in drug solutions. While this approach demonstrated proof-of-concept, it was limited by rapid burst release and short drug delivery duration. Subsequent advances in materials science and nanotechnology have enabled the development of increasingly sophisticated drug-loading and delivery systems. These approaches include molecular imprinting and layer-by-layer assembly, and the incorporation of colloidal and polymeric nanoparticles has facilitated controlled and sustained drug release while preserving optical clarity, oxygen permeability and wearer comfort, while minimising adverse effects on the ocular surface. Similarly, micelles, microemulsions and solid nanoparticles have been employed to achieve controlled release kinetics [19–22]. Challenges associated with drug delivery via contact lenses have been comprehensively summarised [23].

A wide range of drug-eluting contact lenses have been evaluated in pre-clinical studies, including those delivering antibiotics, anti-inflammatory agents and IOP-lowering drugs (Table 1). Studies consistently provide detailed *in vitro* characterisation, including drug incorporation techniques and release kinetics, reflecting substantial progress in contact lens design and development. Cytotoxicity testing and cell culture assays are also commonly reported, supporting biocompatibility assessments.

**Table 1:** Summary of drug-releasing contact lenses studies reporting in vivo results from animal models.

Type of CL and condition	Primary CL	Comparators	Drug loading technique	Pharmacodynamics	Pharmacokinetics
**Bacterial keratitis**					
Kuang et al. [57]	(i) MOX-loaded CL	(ii) MOX eye drops (iii) No treatment	Drug-polymer film in the periphery of the CL	MRSA load and clinical score: CL < eye drops = no treatment AH protein and corneal IL-8: CL < eye drop = no treatment Draize test (i): negative o Cornea structure at Day 3: (i) = (iii)	*C*_max_ in cornea: (i) @2 h, (ii) @0.5 h *C*_max_ in serum (i) @4 h, (ii) @0.5 h. Absorption in cornea, iris, sclera, lens, choroid, retina, AH @18 h: (i) > (ii)
Wang et al. [58]	(i) LEV-loaded CL	(ii) CL soaked in LEV (iii) CL with NPs soaked in LEV (iv) pHEMA CL	Nanoparticle-loaded CL	Efficacy against *S. aureus* and *E. coli*: (i) > (iv) Bacterial keratitis treated with (i): Improvement in clinical signs Day 3 onwards with (i) No pathological change to corneal epithelial and stromal structure with 7 days of (i) wear	Corneal cell viability @72 h: 98% In vitro drug release: (i): 84% release over 120 h (ii): 78% over 10 h (iii): 96% over 5 h
Gade et al. [59]^a^	(i) MOX and DEX-loaded CL	(ii) MOX embedded CL (iii) MOX in solution (iv) DEX embedded CL (v) DEX in solution		Efficacy against *S. aureus* corneal anti-bacterial activity: (ii) > (iii) MIC of (i): >10 μg/g in 1 h	MOX conc in cornea: (ii) > (iii) MOX levels in sclera, lens, AH, VH, plasma: (ii) ≈ (iii)
**Anti-inflammatory**					
Maulvi et al. [21]	(i) DEX-loaded micelle CL	(ii) CL soaked in DEX solution (iii) DEX eye drop	Mixed micelles	Tear IL-6 levels: Similar between (i) and (ii); (i) < (iii) No ocular toxicity noted with (i)	Tear *C*_max_: (i) @1 h ≈ (iii) @15 min < (ii) @4 h Eye drop > control CL > Test CL Mean residence time and AUC_0-24 h_: (i) > (ii) > (iii)
Toffoletto et al. [60]	(i) DEX + peptide loaded CL	(ii) DEX-loaded CL	Peptide	No corneal abrasion or epithelial cell loss after 6 h with (i) and (ii) Reversible swollen vacuoles in corneal epithelial cells with (i) and (ii)	Drug accumulation: Cornea and AH: (i) > (ii) VH and Sclera: (i) ≈ (ii)
Ross et al. [61]	(i) DEX-polymer film-CL	(ii) DEX eye drops (iii) Vehicle (CL + film, without drug) (iv) Intravitreal DEX	Drug-polymer film in the periphery of CL	AUC Fluorescence post-retinal vascular leakage: (i) ≈ (ii) ≈ (iv) < (iii) Conc _protein VH_: (i) ≈ (ii) ≈ (iv) < (iii)	Cornea, sclera, AH, iris, retina conc: (i) > (ii) Serum *C*_max_: (i) @8 h < (ii) @1 h Change in corneal thickness: (i) ≈ (iii) Change in IOP: (i) > (iii) Change in weight: (i) ≈ (iii) Bullae and vacuole epithelial change with (i) Major organ pathology: No change in body weight or pathology with (i)
Bengani et al. [62]	(i) DEX-polymer film-CL	(ii) DEX eye drops (iii) Vehicle (CL + film, without drug)	Drug-polymer film in the periphery of CL	Efficacy against CNV @Days 5 and 7: (i) ≈ (ii) < (iii) Efficacy against induced anterior uveitis: (i) ≈ (ii) CD45+ infiltration: (i) ≈ (ii) Draize test: negative	*C*_max_ in AH: (i) @3 h > (ii) @1 h Change in IOP @Day 7: (i) ≈ (ii) ≈ (iii) Change in corneal thickness @Day 7: (i) < (ii) < (iii) Corneal staining @Day 7: (i) ≈ (ii) ≈ (iii)
DiPasquale et al. [63]	(i) Bromfenac extended-release CL	(ii) Bromfenac eye drops	Polymer with macromolecular memory	After 8 days of (i): no corneal deposits, oedema or vascularisation. (−) uveitis, fundus abnormalities (−) corneal injury, adverse effects on corneal layers or keratocytes, apoptosis	Tear *C*_max_: (i) @Day 7 ≈ (ii) @0.5 h Mean residence time: (i) > (ii) Bioavailability (AUC_0–8 days_): (i) > (ii)
**Glaucoma**					
Bebawy et al. [64]	(i) Atorvastatin and Ginger-derived exosome CL	(ii) Atorvastatin and Ginger-derived exosomes eye drops (iii) Atorvastatin drops		IOP reduction: (i) > (ii) > (iii) TNF-α levels in AH: (i) < (ii) < (iii) IL-6 levels in AH: (i) < (ii) < (iii)	-^α^
Xu et al. [20]	(i) Brimonidine contact lenses	(ii) Microemulsion-laden contact lens (iii) direct brimonidine-loading (iv) soaking method	Silica nanoparticles	Time to IOP reduction: (i) < (iv) Extent of IOP reduction: (i) > (iv)	Release duration: (i) > (ii) > (iv) *C*_max_ released: (i) > (ii) > (iv)
Yang et al. [65]	(i) Timolol and brinzolamide loaded CL	(ii) Blank CL (loaded with ionised water) (iii) Timolol and brinzolamide eye drops (iv) Saline	Micelle	Bioavailability: (i) > (iii) IOP reduction @24 h: (i) ≈ (iii) > (ii) ≈ (iv) IOP reduction @72 h: (i) > (ii) ≈ (iii) ≈ (iv) Draize test: mild irritation with (i) and (ii)	Bioavailability: (i) > (iii) *C*_max_ : (iii) > (i) Time to *C*_max_: (i) > (iii)
**Other**					
Pereira-da-Mota et al. [66]	(i) Pravastatin CL	(ii) Pravastatin eye drops	Soaking	Drug not detected after wearing (i) for 10 h and removed for 14 h No ocular irritation with (i) or (ii)	Tear *C*_max_: (i) @30 min < (ii) @5 min Mean residence time (i) < (ii) Ocular biodistribution: Cornea ≈ AH > sclera Cornea ≈ AH > VH
Sharma et al. [67]	(i) Nintedanib CL with Vitamin E	(ii) Nintedanib eye drops	Loaded with Vitamin E and soaking in drugs	CNV @Day 14: (i) < (ii) Corneal staining: (i) ≈ (ii) Corneal fibrosis severity: (i) < (ii) Time to corneal fibrosis: (i) < (ii)	Conjunctival ingrowth: (i) < (ii) ≈ (iii) Corneal thinning: (i) ≈ (ii) ≈ (iii)
Fu et al. [68]	(i) Atropine eluting CL	(ii) Atropine sulphate eye drops		Efficacy in myopia control: (i) ≈ (ii) Pupil diameter: (i) < (ii) Draize test: Negative (−) corneal inflammation, scleral thickness Corneal fibroblasts unaffected	-^α^

α = No in vivo data available.

*AH* aqueous humour, *AUC* area under curve, *CD45* cluster differentiation marker 45, *CL* contact lens, *C*_*max*_ maximum concentration, *Conc* concentration, *CNV* corneal neovascularisation, *DEX* dexamethasone, *IOP* intraocular pressure, *LEV* levofloxacin *MIC* minimum inhibitory concentration *MOX* moxifloxacin, *MRSA* methicillin-resistant *Staphylococcus aureus*, *NPs* nanoparticles, *pHEMA* polyhexylmethylmethacrylate, *TNF* tumour necrosis factor, *VEGFA* vascular endothelial growth factor A, *VH* vitreous humour.

^a^In vivo data only available for (ii).

However, what is currently lacking is consistent, statistically robust comparisons of the efficacy of drug-delivering contact lenses to standard care or clinically appropriate controls. Instead, efficacy is sometimes inferred from comparisons with pre-treatment baselines or non-therapeutic controls, limiting meaningful interpretation of clinical relevance. The pharmacokinetic and pharmacodynamic findings reported vary across studies, with varying durations and sites of drug accumulation being studied, as well as the choice of clinical outcome measures. Serum and tear concentration, temporal assessment of ocular and systemic biodistribution and clinical and histopathological analysis of drug delivery sites are important for advancing the field toward clinical translation.

Despite positive outcomes in pre-clinical studies, clinical translation of drug-eluting contact lenses has been limited. To date, the only drug-delivering contact lens to gain regulatory approval is Johnson & Johnson’s Theravision, a daily disposable lens containing 19 µg of ketotifen, approved by the FDA in 2022. Randomised controlled trials demonstrated significant reductions in ocular itching and redness following allergen challenge, with efficacy lasting up to 12 h [24]. Another group in Japan reported a retrospective study with Theravision [25]. Patients with seasonal allergic conjunctivitis chose either standard daily disposable lenses (*n* = 17) or Theravision lenses (*n* = 7) for use during pollen season. While overall symptom scores did not differ significantly, initiation of rescue anti-allergy eye drops was modestly delayed in the Theravision group. Despite these promising results, Theravision was discontinued globally in 2024. Although no official reason for its discontinuation was released, limited commercial viability, high manufacturing costs and reduced market uptake may have contributed. This highlights the importance for developers of novel drug-delivery contact lens systems to understand market demand and clinical need, while also investing in practitioner education and training to support adoption and increase uptake.

Contact lens drug delivery designs have also inspired another concept of ocular surface drug delivery through ocular surface patches. One example is a 3D-printed mucoadhesive patch made of polyvinyl alcohol (PVA) and hyaluronic acid, designed for the treatment of bacterial keratitis [26]. The patch measured 15 mm in diameter and 0.3 mm in thickness and was loaded with 300 μg of tobramycin. It demonstrated good optical transparency, significant biofilm inhibition and sustained drug release over 24 h, outperforming conventional tobramycin eye drops in release duration. *In vivo* studies in a rabbit model of bacterial keratitis showed that corneal healing was superior in animals treated with the tobramycin-loaded patch compared to those receiving tobramycin eye drops. Histopathological analysis revealed reduced corneal infiltrates and lower inflammatory cytokine expression in the patch-treated group, indicating enhanced therapeutic efficacy and anti-inflammatory effects. Another patch design incorporated water-soluble microneedle tips loaded with anti-bacterial nanoparticles and a separate epidermal growth factor layer [27]. The microneedle tips rapidly dissolve on lens wear, delivering antimicrobial nanoparticles that significantly reduce bacterial burden. Concurrent release of epidermal growth factor accelerated corneal epithelial regeneration and wound closure. *In vivo* treated eyes showed marked reductions in corneal inflammation, stromal infiltration and opacity, along with improved epithelial healing compared with controls. Clinical testing of such patches for ocular infections is an exciting prospect for future research.

## Biomonitoring Using Contact Lenses

### Intraocular Pressure Monitoring Contact Lenses

The most widely studied commercial contact lens sensor to date is the Triggerfish® contact lens (Sensimed, sensimed.ch/sensimed-triggerfish). It incorporates circumferential strain gauges to detect changes in corneal curvature. Importantly, Triggerfish does not measure absolute IOP in mmHg; instead, it yields an electrical signal expressed in volts or millivolt equivalents relative to an initial baseline measurement. Its approval by the FDA in 2016 was therefore limited to the identification of diurnal IOP-related patterns, rather than an output of numeric IOP values [28]. In Europe, the approval was based on the ability to provide continuous recording of IOP-related dimensional changes. This fundamental limitation has subsequently driven research efforts aimed at translating CL sensor electrical outputs into clinically interpretable IOP measurements. In 2020, a similar pressure-measuring contact lens capable of 24-h IOP monitoring was reported. An early-phase study involving eight participants demonstrated its ability to measure IOP; however, its accuracy and lag in detecting and quantifying IOP fluctuations remain unclear [29].

Since its introduction, Triggerfish has been widely used to investigate 24-h IOP-related patterns in a range of clinical contexts, including patients undergoing cataract surgery, individuals receiving medical or surgical treatment for glaucoma and studies examining the impact of daily activities on IOP variation [30–32]. It has also been explored as a potential tool for predicting glaucoma progression based on 24-h IOP-related data. However, results in this area have been equivocal. In one study published in 2024, only a lower standard deviation of ocular pulse amplitude was found to be predictive of glaucoma progression, although the underlying mechanism for this association remains unclear [30]. The limited uptake is likely to be due to these interpretation challenges, high cost and lack of absolute IOP measurement.

More recently, several studies have used calibration strategies to convert electrical signals from CL sensors into estimates of absolute IOP, incorporating the known effects of body position on IOP [31]. This approach has since been advanced to calibrate for postural changes in IOP. Applanation tonometry is conducted in sitting and supine positions, or at incremental head angle variations of approximately 10 degrees, followed by repeat CL sensor measurements in the same positions to develop signal–IOP conversion models. One such model in 2024 was evaluated in a cohort of 80 participants, including healthy individuals and patients with primary open-angle glaucoma across a range of IOP values, with predicted IOP measurements generally within ±3 mmHg of tonometric values obtained from the fellow eye [32]. A similar calibration approach in 2025 was employed in a study involving 59 participants who wore a CL sensor continuously for 24 h, in which IOP measurements at multiple head positions before and after lens wear were used to train a signal-to-pressure conversion model. While this study demonstrated the feasibility of long-term IOP monitoring and electrical signal-to-IOP conversion, the absence of a comparator group during continuous CL wear limited conclusions regarding accuracy, relative to standard tonometric techniques.

Other more recent advances have focused on nanomaterial-enhanced strain sensors, particularly using two-dimensional materials such as MXene. MXene materials are lightweight, flexible and can be spray-deposited, making them suitable for use in wearable contact lenses [33]. The high electrical sensitivity of MXene-based sensors enables the detection of small IOP changes. One CL sensor incorporating MXene allowed real-time, wireless transmission of IOP-related signals to a smartphone interface, addressing key limitations of earlier devices. Biocompatibility was confirmed in human umbilical vein endothelial cells and *in vivo* testing in rabbits, which revealed no corneal epithelial damage with fluorescein [34]. These findings support the ocular safety of MXene-integrated lenses while highlighting their potential for continuous, high-sensitivity monitoring.

Perhaps, the most advanced iteration of contact lens-based IOP monitoring is exemplified by a recently published neuroprosthetic smart contact lens system [35]. This platform integrates an ultra-high-sensitivity MXene-based strain sensor with a neural interface, enabling not only continuous IOP sensing but also sensorimotor feedback. In rat models, abnormal IOP was detected as an electrical signal in the contact lens and transmitted to the somatosensory cortex, eliciting graded neural responses that were subsequently processed by the motor cortex, resulting in observable muscle movements, such as leg twitching. This closed-loop system represents a conceptual shift from passive monitoring toward active physiological feedback, suggesting future applications in automated alerts or therapeutic interventions.

### Tear Glucose Monitoring

Over the past two decades, tear glucose-monitoring contact lenses have been developed using a range of sensing strategies, including fluorescence-based systems, nanomaterial-enhanced sensors, near field communication (NFC)-enabled transmission and specialised micro-electrodes [36–40]. Among these approaches, electrochemical biosensors are widely used, with glucose-mediated chemical reactions generating measurable electrical signals. Additionally, one group has demonstrated retinal drug delivery through monitoring contact lenses, suggesting future applications in diabetic retinopathy beyond glucose monitoring [41]. The recent systematic review by Yazdani et al. provides an up-to-date overview of these advances, highlighting current prototypes, their efficacy and remaining challenges [42].

Most devices have been evaluated in small animal studies, with limited safety data. Reported outcomes typically include correlation between tear glucose and blood glucose, lag time and analytical accuracy. Across studies, correlation coefficients in animal models have improved markedly over time, increasing from approximately 0.76 in 2020 to values as high as 0.96 in 2024, meeting accepted performance thresholds for new glucose-monitoring technologies [38, 39]. One study is notable as the only work to include human testing [38], where a soft contact lens was developed with an electrochemical glucose biosensor and an NFC (Near Field Communication) chip for wireless transmission of tear glucose data to a smartphone. In a pilot study of 10 individuals with diabetes and 10 non-diabetic controls, strong correlations were observed between tear glucose and blood glucose (*r* > 0.91 in healthy participants and >0.94 in diabetic participants), with lag times of approximately 8–15 min. These lag times were comparable to those reported in animal models, although correlation coefficients were modestly lower in humans, suggesting additional physiological or environmental factors influencing tear glucose dynamics. Notably, the study highlights the need for individualised calibration, as inter-individual variability in glucose diffusion into the tear film necessitates personalised lag-time adjustment for accurate longitudinal monitoring.

## Contact Lenses for Visual Augmentation

### Accommodating Soft Contact Lenses

Accommodating smart soft contact lenses represent an emerging approach for presbyopia correction. Unlike conventional multifocal designs, which rely on simultaneous distance and near zones and depend on pupil size changes, these advanced lenses aim to adjust optical power dynamically in response to the user’s viewing conditions. By detecting eye orientation and viewing distance, they can modulate their refractive power to match the accommodative demand in real time [43].

The mechanisms of this adaptability have been described to date in patent filings and summarised here from Morgan et al. [43]. Some designs use mechanically driven fluid reservoirs within the lens, where subtle eye and eyelid movements alter the lens shape and, consequently, its optical power. Others incorporate electronic components, including sensors that detect viewing distance and angle, paired with actuators that reshape the lens to adjust focus. Hybrid approaches have also been explored, combining mechanical systems with electro-sensitive elements; in these designs, electrochemical sensors detect changes in viewing distance and trigger fluid redistribution within the lens, resulting in a controlled change in lens curvature and refractive power. While the evidence in clinical use is currently limited, this would be a major step in expanding the refractive application of contact lens wear and retaining ageing wearers in contact lens wear.

### Higher-Order Aberration Correcting Contact Lenses

Higher-order aberrations significantly degrade visual quality, particularly in individuals with corneal diseases such as keratoconus [23]. Standard soft contact lenses that conform to the anterior corneal shape are unable to correct these aberrations, while rigid lenses can provide correction. However, residual aberrations from the posterior corneal surface and/or crystalline lens may manifest once the anterior corneal surface is neutralised with a rigid lens. Wavefront-guided scleral lenses can reduce these residual higher-order aberrations, leading to improvements in visual acuity of between 0.01-0.03 logMAR as well as contrast sensitivity [44–46]. Despite these advances, it remains unclear whether such improvements in measured high contrast visual acuity translate into improved vision in daily life.

### Augmented Reality Contact Lenses

Augmented reality contact lenses represent an ambitious extension of smart contact lens technology, aiming to integrate sensing, computation and visual display within a form factor compatible with wear. Early high-profile efforts included the Google–Novartis collaboration on a smart contact lens designed to monitor glucose levels in tears. This project was ultimately discontinued after it concluded that tear glucose concentrations could not be correlated reliably with serum glucose levels, limiting clinical utility [47]. However, this contrasts with other emerging research findings, which show a strong correlation between tears and blood glucose [38, 42]. It is unclear whether commercial considerations, rather than solely scientific limitations, contributed to the decision to discontinue the project.

More recently, Mojo Vision (mojo.vision/) emerged as a leading developer of augmented reality contact lenses incorporating microLED displays, motion sensors, radios, processors, gyroscopes and magnetometers within the lens architecture [48]. Despite producing several functional prototypes, the device was not approved for wear, and the company subsequently pivoted toward other avenues. Limitations included the need for an external wearable device to enable wireless communication and incompatibility with existing consumer smartphones. In parallel, companies such as InWith (inwithcorp.com) have focused on developing enabling technologies that embed circuitry within hydrogel contact lenses, positioning themselves as platform providers to support future smart and augmented reality contact lens development rather than delivering end-user devices [49]. Collectively, these efforts reflect rapid technological progress and the ambitious scope of augmented reality contact lens development, extending beyond conventional wearables, while underscoring substantial technical, physiological and regulatory challenges to clinical and commercial translation.

### Photochromatic Contact Lenses

Photochromatic contact lenses were first introduced in 2018 following FDA approval of senofilcon A lenses (Acuvue Oasys with Transitions; Johnson & Johnson Vision, jnjvisionpro.com/en-us/). These lenses can block up to 70% of visible light when activated by ultraviolet exposure. Company-sponsored clinical trials reported improvements in photostress recovery, glare disability, glare discomfort and chromatic contrast when compared with the fellow eye without a contact lens [50]. In comparison with standard clear lenses, users also reported a preference for photochromatic lenses during daytime driving and outdoor activities [51]. Despite such reported benefits, Acuvue Oasys with Transitions was discontinued in 2024 [52], possibly due to limited consumer uptake, leaving no commercially available photochromatic contact lens options currently on the market.

### Contact Lenses for Colour Vision Deficiency

Chromagen lenses (chromagen.us), which comprise a set of eight tinted soft lenses, have been available on the market for several decades. Although they can improve performance on colour vision tests, reductions in visual acuity may occur due to decreased light transmittance associated with the tint [53, 54].

## Future Research Directions

Future research in the contact lens field should not only focus on advancing the technology, but also on improving clinical translation, confirming long-term safety and real-world usability. Many contemporary contact lens technologies for drug delivery remain limited to proof-of-concept or small pre-clinical studies, with comparatively few adequately powered human clinical trials evaluating long-term efficacy, safety, comfort and wearer adherence. Standardisation of outcome measures, particularly in drug delivery and biosensing studies, would improve comparability between studies and support regulatory approval pathways. For myopia control applications, further longitudinal studies are needed to characterise long-term ocular health outcomes, rebound effects following treatment cessation and optimal strategies for minimising discontinuation. Optimisation of myopia control effects through a combination of optical design and pharmacological interventions is an intriguing approach for the future.

Advances in materials science, nanotechnology, wireless communication and miniaturised electronics are likely to accelerate the development of smart contact lenses capable of simultaneous sensing, monitoring and therapeutic intervention. However, successful commercialisation will depend on demonstrating clear clinical benefit over existing alternatives while maintaining affordability, comfort and ease of use. Therefore, future innovation will require multidisciplinary collaboration between clinicians, engineers, material scientists, regulatory bodies and industry to ensure that emerging technologies address genuine clinical needs and achieve meaningful patient uptake.

Patient perspectives of contact lens-based drug therapies are reported infrequently. Two studies have reported similarly that about 1 in 3 patients with glaucoma would accept contact lens drug delivery systems, with more favourable support when they had the potential to avoid glaucoma surgery and less favourable support when lenses were proposed primarily as substitutes for topical eye drops [55, 56]. Therefore, further mixed-methods research incorporating patient, clinician and industry perspectives is needed to guide development priorities within the rapidly expanding field of contact lenses as drug-delivery technologies. Importantly, robust clinical evidence and long-term safety data will ultimately determine whether these technologies can achieve sustained clinical adoption.

Similarly, for contact lenses targeting infectious and inflammatory ocular diseases, it remains important to establish whether sustained drug delivery and prolonged ocular surface residence translate into clinically meaningful improvements, such as faster recovery, reduced dosing burden and fewer adverse effects, including corneal scarring. Addressing these questions through well-designed comparative clinical studies will be essential to determine the true clinical value of these emerging technologies. Cost of production is also likely to be a major determinant of clinical translation in this area. Many experimental drug-delivery systems rely on sophisticated nanotechnology platforms, specialised fabrication techniques or advanced material engineering processes that may be difficult or expensive to manufacture at a commercial scale. Although some of these technologies may become commercially viable in the future with applications in other industries, it is currently unclear which approaches are most likely to succeed in advanced contact lens applications.

## Summary

This review reflects the rapid recent development of contact lens technologies beyond refractive correction in myopia control, drug delivery, sensing of systemic or ocular diseases and ocular theranostics, visual enhancement or augmentation and the potential impact of the digital transformation in these applications. Translation and commercialisation of these technologies have so far been limited, with the exception of contact lenses for myopia control and some limited sensing applications, but there is enormous potential for personalised disease diagnosis and treatment and optical enhancements in the next 10–20 years.
